# Relative Importance of Coral Cover, Habitat Complexity and Diversity in Determining the Structure of Reef Fish Communities

**DOI:** 10.1371/journal.pone.0083178

**Published:** 2013-12-13

**Authors:** Valeriya Komyakova, Philip L. Munday, Geoffrey P. Jones

**Affiliations:** ARC Centre of Excellence for Coral Reef Studies, and School of Marine and Tropical Biology, James Cook University, Townsville, Queensland, Australia; Centre National de la Recherche Scientifique, France

## Abstract

The structure of coral reef habitat has a pronounced influence on the diversity, composition and abundance of reef-associated fishes. However, the particular features of the habitat that are most critical are not always known. Coral habitats can vary in many characteristics, notably live coral cover, topographic complexity and coral diversity, but the relative effects of these habitat characteristics are often not distinguished. Here, we investigate the strength of the relationships between these habitat features and local fish diversity, abundance and community structure in the lagoon of Lizard Island, Great Barrier Reef. In a spatial comparison using sixty-six 2m^2^ quadrats, fish species richness, total abundance and community structure were examined in relation to a wide range of habitat variables, including topographic complexity, habitat diversity, coral diversity, coral species richness, hard coral cover, branching coral cover and the cover of corymbose corals. Fish species richness and total abundance were strongly associated with coral species richness and cover, but only weakly associated with topographic complexity. Regression tree analysis showed that coral species richness accounted for most of the variation in fish species richness (63.6%), while hard coral cover explained more variation in total fish abundance (17.4%), than any other variable. In contrast, topographic complexity accounted for little spatial variation in reef fish assemblages. In degrading coral reef environments, the potential effects of loss of coral cover and topographic complexity are often emphasized, but these findings suggest that reduced coral biodiversity may ultimately have an equal, or greater, impact on reef-associated fish communities.

## Introduction

Coral reefs are well-known for the high diversity of fishes that are closely associated with the reef substratum [1–4]. Reef fishes exhibit diverse patterns in their association with coral reef habitat, relying on different substrata for critical resources such as food, shelter and living space. Many studies have indicated that both coral cover [5–10] and topographic complexity [11–16] are particularly important in explaining local reef fish diversity and abundance. In the context of reef degradation, the effects of loss of coral cover and declining topographic complexity on reef fish biodiversity have been widely emphasized [9,10,17–20]. However, reef structure and the effects of degradation potentially involves a wide range of structural features, including coral cover, topographic complexity, coral diversity and different coral growth forms [9,21–24]. In general, the importance of declining coral diversity and the relative importance of the different structural changes to reef habitat are poorly understood. 

The species richness and abundance of reef fish communities have often been related to structural or topographic complexity – a measure of variation in the vertical relief of the habitat (e.g. [11,12,14–16,25]). High topographic complexity may promote high abundance and diversity because it provides more refuges, decreases the encounter rates between competitors as well as between predators and their prey, and consequently reduces the effects of predation and competition [26–30]. High complexity may also promote the co-occurrence of the species that are specialised on different reef habitat features, such as vertical walls, horizontal reef matrix and caves [31]. Although habitat topographic complexity has been identified as one of the major predictors of coral reef fish diversity, it does not appear to be universally important (e.g. [32,33]), and the possibility that it co-varies with other important habitat characteristics has rarely been considered. 

A large number of studies have shown a positive relationship between fish diversity or abundance and live coral cover (e.g. [5–9]). Coral cover is expected to be particularly important in explaining the abundance of obligate coral-dwelling species [34,35], corallivorous fishes [5,36–38] or species reliant on coral habitat for recruitment [9,13,39,40]. Declines in the abundance of these coral-dependant species have often been associated with loss of coral cover [9,10,19,41,42]. However, declining coral cover may or may not be associated with other factors such as loss of coral species and topographic complexity, which makes assessing the overall significance of coral loss difficult. 

The observed differences in the effects of coral cover or topographic complexity on fish abundance and diversity may also be explained by changes in habitat diversity. A number of studies have shown positive relationships between fish community structure and habitat diversity (e.g. [5,7,8]), coral diversity and coral species richness (e.g. [5,7,8]), while others have not found these relationships (e.g. [11,12,32]). The conflicting results may stem from the use of different habitat diversity measures. Several studies interpret habitat diversity as total diversity of all habitat types present (e.g. [11,32,33]), while others focus just on coral diversity (e.g. [7,8]). Some assess diversity using species richness measures (e.g. [8]), while others use diversity indices of different kinds [7,8,11,32,33]. Furthermore, Chabanet et al. [8] pointed out that the relationship between fish diversity and the diversity or abundance of corals may be distorted in the literature due to difficulties associated with classifying corals to species. 

There are a number of reasons to expect that a reduction in coral diversity will affect reef fish communities. Most reef fish species have specific microhabitat requirements [4,31,43] and many are highly dependent on a narrow suite coral species [1,2,34,44] or coral morphology [45–47] for shelter or reproduction sites. However, because coral species differ greatly in their growth form and branching structure [48], coral diversity and reef topographic complexity may be closely linked. This makes it difficult to assess the relative importance of different habitat variables in structuring fish communities.

Our understanding of the relative importance of topographic complexity, coral cover and habitat diversity has been hampered by the limited number of studies that have addressed all these factors together. Also, because these habitat variables are potentially interrelated, statistical techniques that can isolate the contribution of each factor are required. The aim of this study was to investigate the relationships between small site-attached reef fish species richness, total abundance and species composition, and a range of habitat characteristics (structural complexity, habitat diversity, coral diversity, and coral species richness, percent hard coral, branching and plate coral cover). Regression tree analysis and distance-based linear models were applied to quantify the variance in fish community structure explained by the different habitat factors and determine thresholds in these variables at which the greatest changes in reef fish communities occurred. This assessment aimed to investigate what kinds of changes to reef health will have the greatest impact on coral reef fishes, and at what thresholds of habitat decline can the greatest losses of fish biodiversity be expected.

## Materials and Methods

### Ethics Statement

This study was conducted in accordance with Great Barrier Reef Marine Park Authority requirements for non-extractive research and was compliant with James Cook University Code of Conduct for Research in the Great Barrier Reef. An authorisation for this limited impact, non-extractive research in the Great Barrier Reef Marine Park was obtained from James Cook University (Authorisation letter number: MBA5). This research did not involve any endangered or protected species and no animals were sampled. This study was conducted in compliance with the James Cook University Ethics Review Committee regulations (Ethics approval project number: A1124). 

### Study Location

Sampling was conducted within the lagoon of Lizard Island, northern Great Barrier Reef (14ᵒ40′ S, 145ᵒ28′ E), in November 2006 to January 2007. The lagoon encompasses an extensive area of protected coral reef. The reef flat is largely degraded due to regular sun exposure at low tides. It is dominated by soft coral, dead coral rubble and partially dead massive corals. The reef edge and slope are dominated by hard corals, predominantly by *Porites cylindrica*. The community of small site-attached reef fishes largely consisted of species from the families Pomacentridae (damselfishes) and Labridae (wrasses).

### Sampling Design

To estimate the effects of a range of habitat characteristics on the diversity and structure of the fish community within the lagoon, twenty-two 2x2 m quadrats were surveyed at each of three sites: Palfrey Island, Lizard Head and Bird Island reef. The sampling methodology used in this study is an adaptation of the previously used techniques [49–55]. The sites were selected haphazardly within the lagoon, where extensive reefs with a range of habitat types were present. Lizard Head and Bird Island reefs had more extensive reef slopes than Palfrey Island, and potentially are subject to larger current movements due to their proximity to the channel. No other obvious physical differences occur among sites. Quadrats were placed haphazardly on the reef flat and crest at depths ranging from approximately 1-4 m. This sampling effort and quadrat size was considered sufficient to represent the range of habitat diversity and habitat structural complexity present at each site. Quadrats were marked out by placing marker buoys at each of the corners. 

#### Fish measurements

Fish were allowed to become accustomed to the presence of the quadrat markers for 5 min before censuses were conducted. Each quadrat was then observed for 10 min, during which time all fish species present were recorded. During the first 5 min all the larger and more obvious fishes were counted. During the second 5 min, the quadrat was searched for smaller, more cryptic fish species. Fish that entered the quadrat during the 10 min observation period and remained within the quadrat during this time were included in the counts. Fish that swam through the quadrat during the observation period were not counted. All observations were made on SCUBA.

#### Substratum measurements

Topographic complexity of the substratum was assessed for each quadrat using the chain method [11,12]. Tape measures were first placed across the diagonal corners of the quadrat. A fine link chain was then placed underneath each tape measure conforming as closely as possible to all contours and crevices. Topographic complexity was estimated as a ratio of actual surface distance relative to linear distance along each diagonal transects. The average of the two estimates for each quadrat was calculated and used in the analyses.

Habitat within the quadrats was categorized into a number of live coral, dead coral, and other substrates. Live corals were indentified to the lowest possible taxonomic level. Non-live coral was categorized into 16 different substratum types and 11 growth forms were recognised for live and dead corals (Table 1). For each substrate type within each quadrat, percentage cover was calculated based on the total distance each substrate type intercepted each of the two diagonal transects (recorded to the nearest centimetre) as a proportion of the total length of the two transects. The percent cover of non-live coral substratum types, dead coral growth forms and live coral taxa were used to calculate habitat diversity. All coral species present within the quadrat were counted to estimate species richness. Finally, Simpson’s diversity indexes were calculated for habitat and coral diversity.

**Table 1:** Substrate types and hard coral growth forms recognised.

**Substrate types**	**Hard coral growth forms**
Coarse sand	Massive
Fine sand	Arborescent
Coarse coral rubble	Branching
Fine coral rubble	Corymbose/Plate
Macro-algae	Caespitose/Plate
Turf algae	Columnar
Dead coral (including growth form)	Digitate
Dead reef complex	Bottle-brush
*Lobophytum spp.*	Folious
*Sarcophyton spp.*	Encrusting
*Sinularia spp.*	Free-living
Other soft corals	
Clams	
Sponges	
Ascidians	
Rock	

### Statistical Analysis

#### Bivariate relationships

Exploratory analysis of the strength and form of the relationships that fish species richness and total reef fish abundance exhibited in relation to coral species richness, coral cover and topographic complexity were examined using regression analyses. Fish abundance was log_10_ transformed in order to meet statistical assumptions. Given the potential for co-variation in the habitat characteristics, the interrelationships between coral cover, coral species richness and topographic complexity were also examined using correlation analysis. Data from the three sites were pooled for examining these general relationships.

#### Regression tree analysis

A regression tree approach [56] was used to explore and describe the relationship between fish species richness and abundance and a range of environmental variables: site, topographic complexity, habitat diversity (Simpson’s index), coral species richness, coral diversity (Simpson’s index), percent hard coral cover, branching coral cover and corymbose coral cover (Table 2). Corymbose corals were recognised as corals with “horizontal interlocking branches and short upright branchlets" [48]. Corymbose corals have been shown to be a preferred habitat type for some of coral reef fish species [34,57]. Therefore corymbose coral cover may influence total fish species richness and abundance.

**Table 2:** Mean and S.E. of the seven environmental variables.

**Variable**	**Mean +/- S.E.**
Topographic complexity	1.67+/-0.03
Habitat diversity (Simpson's index)	0.71+/-0.02
Coral diversity (Simpson's index)	0.4+/-0.03
Coral species richness	7.17+/-0.49
Hard coral (%)	26.95+/-2.13
Branching coral (%)	10.38+/-1.67
Corymbose coral (%)	3.63+/-0.66

Fish abundance was log_10_ transformed to reduce the influence of extreme values. Analysis was performed using the TreesPlus statistical computer package. Squared deviations were used as split measures for fish species richness and absolute deviations were used as split measures for fish abundance data. The size of each tree was selected by cross-validation, with a four-leaf tree having the smallest estimated predictive error in both cases. Regression tree analysis was used because it is well suited to data sets that are not fully balanced, have missing values or many zero values, and exhibit non-linear relationships between variables and high order interactions [56], such as are common in environmental and biological data sets. 

#### Distance-based linear modelling

Distance-based linear modelling [58,59] was used to further explore the relationship between the nineteen most abundant fish taxa (Table 3) and seven major substrate variables: habitat topographic complexity, habitat diversity, coral diversity, coral species richness, percent hard coral cover, branching coral cover and corymbose coral cover (Table 2). Bray-Curtis distance measures and forward selection of predictor variables were used in the models [58,59]. Distance-based redundancy analysis (dbRDA) was used as a visualisation tool [59]. Both analyses were performed using PRIMER 6 statistical package with PERMANOVA+ add-on (PRIMER-E, Plymouth Marine Laboratory, UK). The abundances of individual taxa were square root transformed. The specific aim of these analyses was to establish habitat types that were occupied or ignored by the most abundant fish taxa, as opposed to establishing dominance patterns in fish community in regards to habitat use.

**Table 3:** Total abundance of fish species used in forward DistLM and dbRDA analyses.

**Species**	**Totals**	**Mean & S.E.**
*Acantochromis polycanthus*	320	4.85 (+/-1.27)
*Amblyglyphidodon curacao*	62	0.94 (+/-0.15)
*Apogon cyanosoma*	80	1.21 (+/-0.34)
Blenniidae	95	1.44 (+/-0.23)
*Chaetodon sp.*	80	1.21 (+/-0.20)
*Chromis viridis*	197	2.98 (+/-0.93)
*Coris batuensis*	49	0.74 (+/-0.17)
*Dischistodus* *sp*.	60	0.91 (+/-0.14)
*Gobiodon sp.*	47	0.71 (+/-0.14)
*Halichoeres melanurus*	93	1.41 (+/-0.15)
Labrids	117	1.77(+/-0.21)
*Plectroglycophidodon lacrymatus*	47	0.71 (+/-0.15)
*Pomacentrus adelus*	43	0.65 (+/-0.14)
*Pomacentrus ambionensis*	70	1.06 (+/-0.29)
*Pomacentrus chrysurus*	44	0.44 (+/-0.25)
*Pomacentrus lepidogenys*	72	1.09 (+/-0.50)
*Pomacentrus moluccensis*	744	11.27 (+/-1.74)
*Pomacentrus wardii*	107	1.62 (+/-0.23)
*Stegastes sp.*	65	0.98 (+/-0.24)

## Results

A total of 115 fish species and 2637 individuals were counted in the 66 quadrats at 3 sites in the lagoon of Lizard Island. Fish communities were dominated by damselfishes, which represented 36% (41 species) of the total number of species encountered, followed by wrasses, which accounted for 16% (18 species).

### Bivariate Relationships between Fish and Habitat Variables

#### Fish species richness

**Figure 1:**
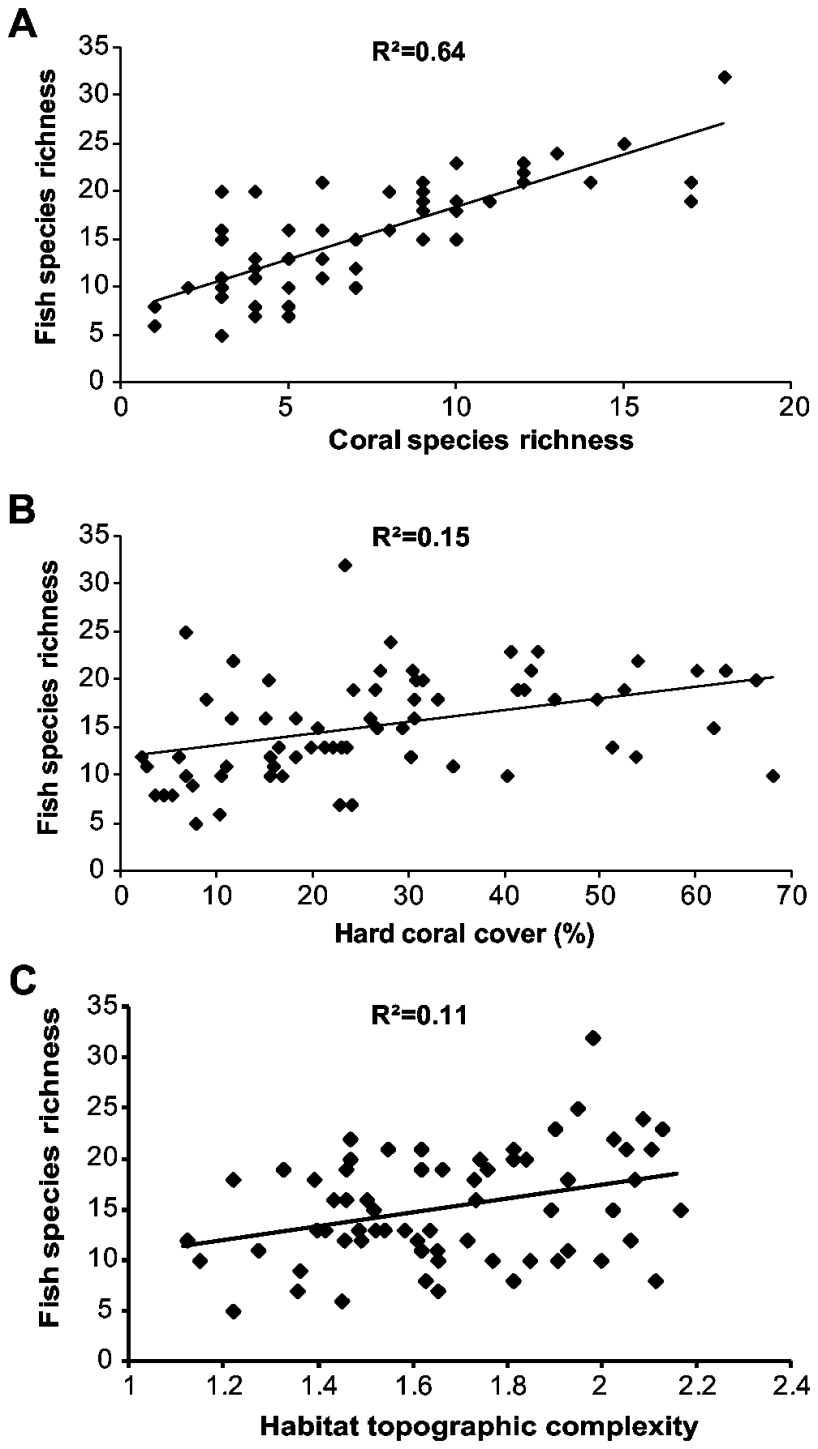
Relationships between fish species richness and habitat variables in the lagoon of Lizard Island, GBR. All relationships were statistically significant (p<0.05).

Fish species richness exhibited a strong linear relationship with coral species richness (Figure 1a, r² = 0.64, *F*_1, 64_ = 114.71, p < 0.001). Fish species richness increased from less than 10 species in quadrats with less than 3 coral species to over 20 species in quadrats with high coral species richness (Figure 1a). Fish species richness was positively related to hard coral cover, but the relationship was weaker (Figure 1b, r² = 0.15, *F*_1, 64_ = 11.47, p < 0.01). Fish species richness was also positively related to topographic complexity, but again accounted for considerably less variation than coral species richness (Figure 1c, r² = 0.11, *F*_1, 64_ = 8.15, p < 0.01).

#### Fish total abundance

**Figure 2:**
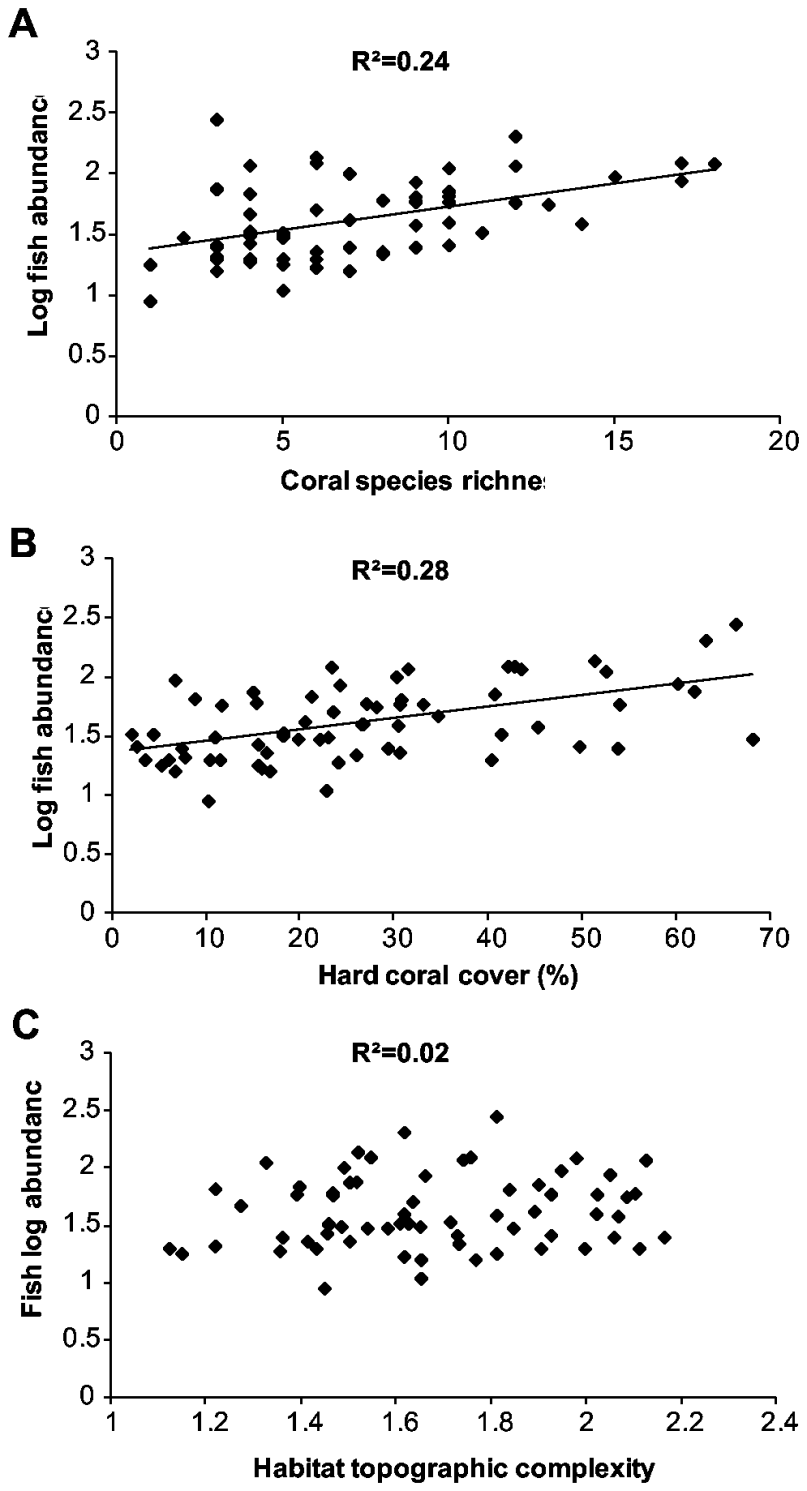
Relationships between log fish abundance and habitat variables in the lagoon of Lizard Island, GBR. Lines were only fitted to the statistically significant relationships (p<0.05).

Log total fish abundance exhibited a significant linear relationship with coral species richness (Figure 2a, r² = 0.24, *F*_(1, 64)_ = 19.64, p < 0.001) and hard coral cover (Figure 2b, r²=0.28, *F*_(1, 64)_ = 24.83, p < 0.001). Coral species richness and hard coral cover explained very similar amounts of the variation in log fish abundance between quadrats (24% and 28% respectively). There was no significant relationship between log fish abundance and habitat topographic complexity (Figure 2c, r²=0.02, *F*_(1, 64)_ = 1.26, p > 0.05).

### Correlations among Habitat Variables

**Figure 3:**
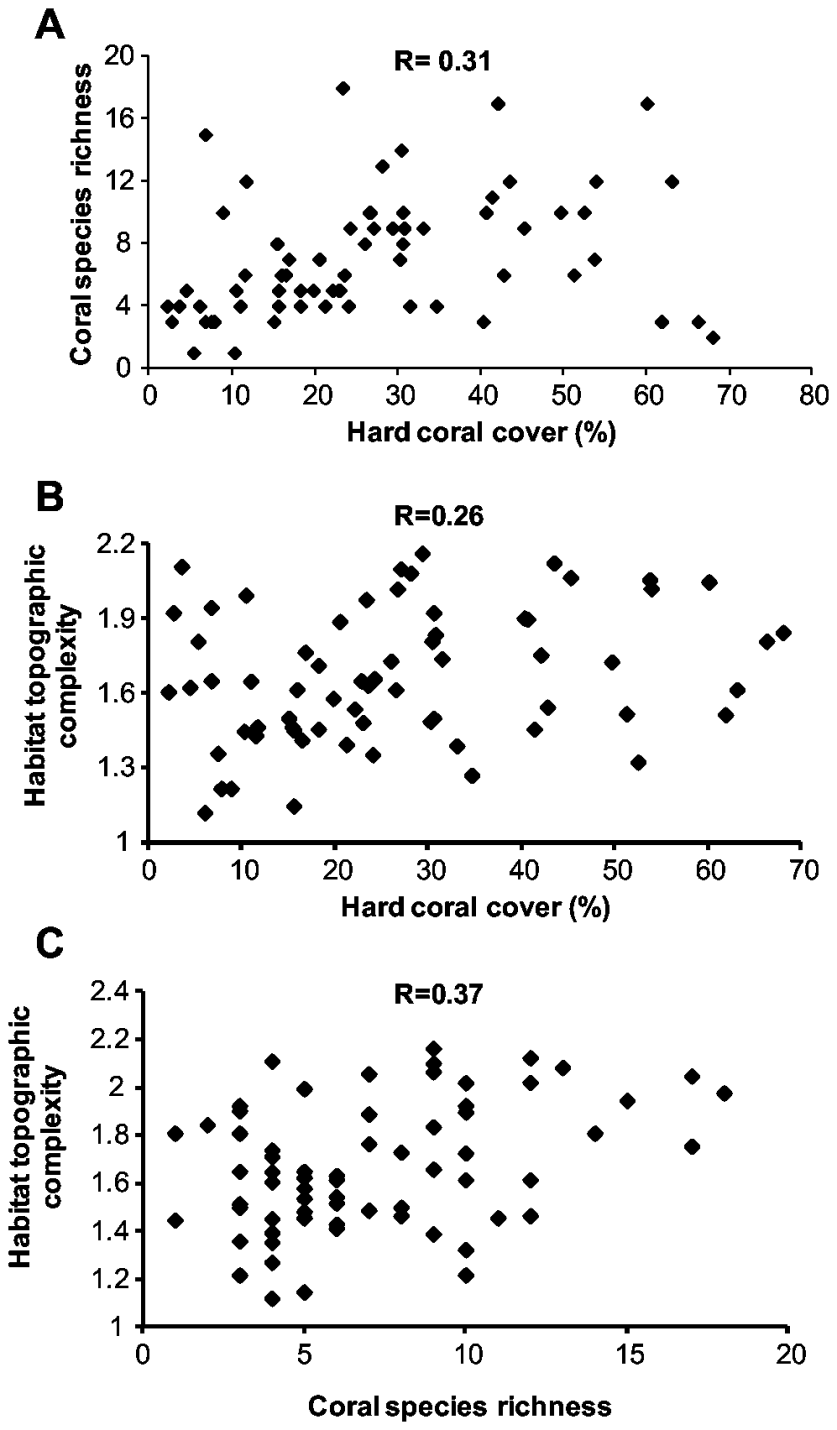
Relationships between habitat variables in the lagoon of Lizard Island, GBR. All relationships were statistically significant (p<0.05).

There was significant co-variation among the three habitat variables: coral cover, coral species richness and topographic complexity (Figure 3). However, in each case, the correlations were relatively weak. Hard coral cover was positively correlated with coral species richness (Figure 3a, r=0.31, p < 0.05) and habitat topographic complexity (Figure 3b, r=0.26, p < 0.05). Habitat topographic complexity was also positively associated with coral species richness (Figure 3c, r=0.37, p < 0.01).

### Regression Tree Analyses - Fish Species Richness and Abundance

#### Fish species richness

**Figure 4:**
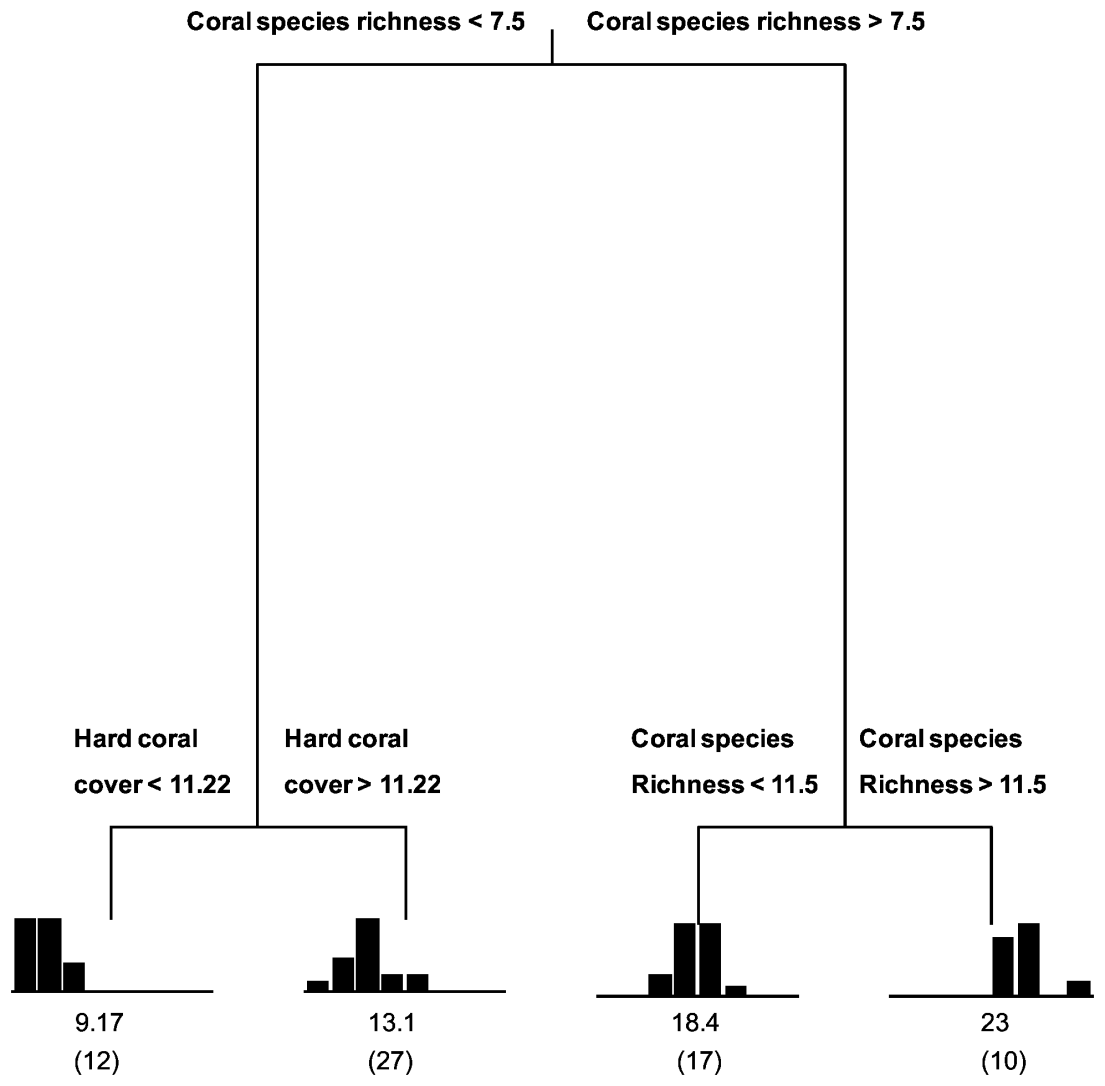
Regression tree analysis of the fish species richness at Lizard Island, QLD, Australia. The explanatory variables were: site, topographic complexity, habitat diversity, coral species richness, coral diversity, percent hard coral cover, branching coral cover and corymbose coral cover. For each of the four terminal nodes the distribution of the observed values of fish species richness is shown in a histogram. Each node is labeled with the mean rating and the number of observations in a group (in parentheses). The tree explained 70.5% of the total variability in the data. The first and second splits based on coral species richness explained 56.4% and 7.2% respectively, the third split based on percent hard coral cover explained additional 6.9%.

Regression tree analysis of the spatial distribution of the fish species richness resulted in a four-leaf tree, explaining 70.5% of the variance among quadrats, with splits based on coral species richness and percent hard coral cover. Overall, coral species richness explained the majority of the variation in fish species richness among quadrats (63.6%), while live coral cover explained an additional 6.9%. No other variables explained significant amounts of variation (Figure 4). 

By and large, there was a positive relationship between fish species richness and coral species richness, with fish species richness being highest in quadrats in which coral species richness was greater than 11.5 (mean = 23, n = 10) (Figure 4) Within the quadrats with lowest coral species richness (i.e. < 7.5) there was an additional split based on the percent hard coral cover, with the lowest fish species richness being in quadrats with hard coral cover lower than 11.3% (mean = 9.17, n = 12). Intermediate fish species richness was observed in quadrats with intermediate coral species richness (mean = 18.4, n = 17) and in quadrats with coral species richness lower than 7.5 and hard coral cover higher than 11.3% (mean = 13.1, n = 27) (Figure 4).

#### Fish abundance and habitat characteristics

**Figure 5:**
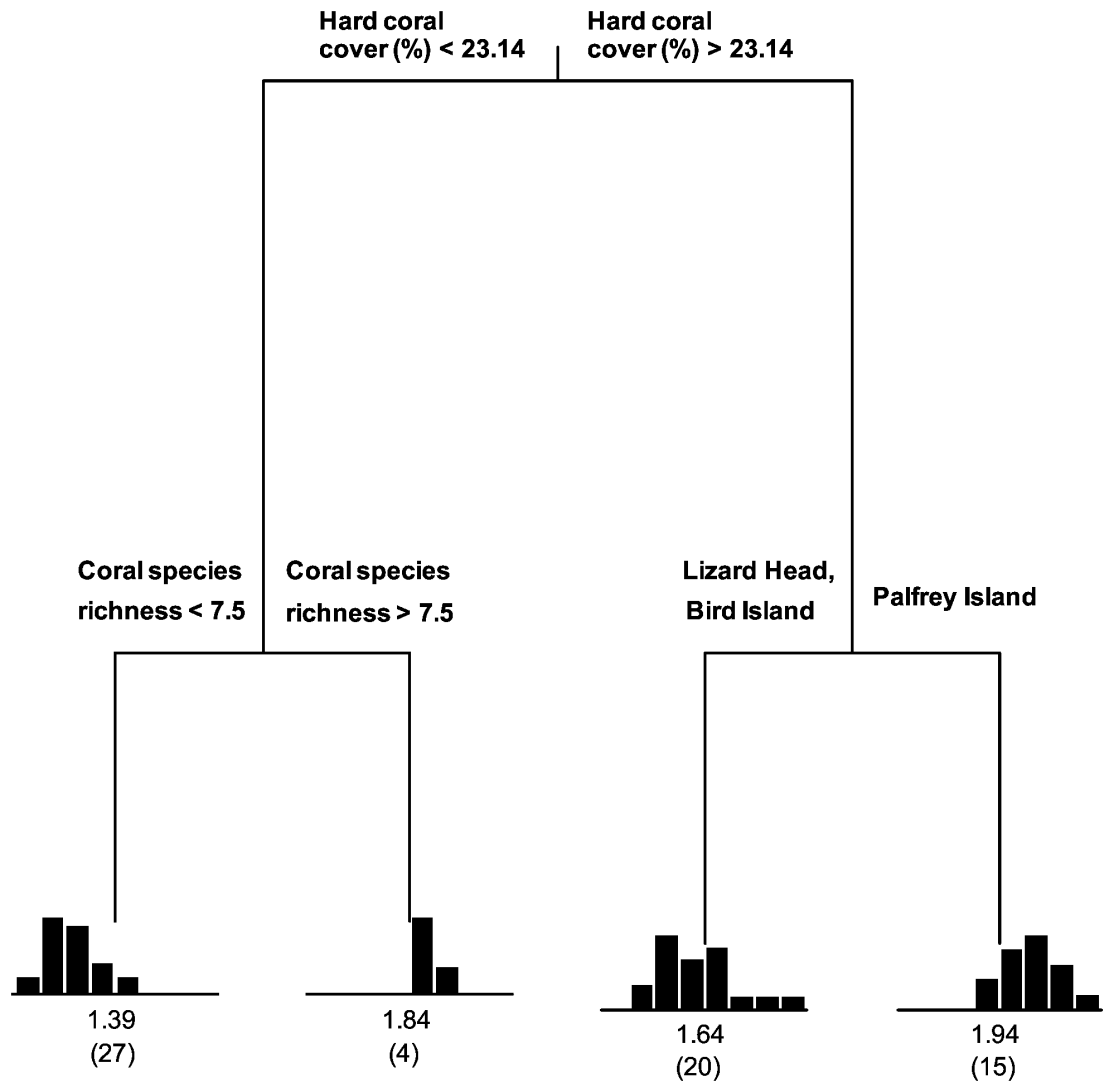
Regression tree analysis of the log fish abundance at Lizard Island, QLD, Australia. The explanatory variables were: site, topographic complexity, habitat diversity, coral species richness, coral diversity, percent hard coral cover, branching coral cover and plate coral cover. For each of the four terminal nodes the distribution of the observed values of log fish abundance is shown in a histogram. Each node is labeled with the mean rating and the number of observations in a group (in parentheses). The tree explained 34.1% of the total variability in the data. The first split based on percent hard coral cover explained 17.4%, second split based on coral species richness explained 8.1% and the third split based on site explained additional 8.6%.

Regression tree analysis of the spatial distribution of the (log) fish abundance produced a four-leaf tree, explaining 34.1% of the variance among quadrats, with splits based on percent hard coral cover, coral species richness and site. Percent hard coral cover explained the majority of the variation in the fish abundance among quadrats (17.4%), while coral species richness and site an explained additional 8.1% and 8.6% respectively. No other variables explained significant amounts of variation (Figure 5).

Overall, there was a positive relationship between fish abundance and percent hard coral cover, with fish abundance being highest in quadrats placed at Palfrey Island in which hard coral cover was greater than 23.14% (mean = 1.94, n = 15) (Figure 5) A positive relationship was also detected between fish abundance and coral species richness, with the second highest fish abundance observed within quadrates in which hard coral cover was lower than 23.14% and coral species richness was higher than 7.5 (mean =1.84, n= 4). Intermediate fish abundance was recorded in quadrats with high hard coral cover (> 23.14%) at Lizard Head and Bird Island (mean = 1.64, n = 20) (Figure 5). The lowest fish abundance was observed within quadrates with lowest hard coral cover (< 23.14%) and lowest coral species richness (< 7.5) (mean = 1.39, n = 27) (Figure 5)

. 

### Distance-Based Linear Modelling

#### Fish community composition

**Figure 6:**
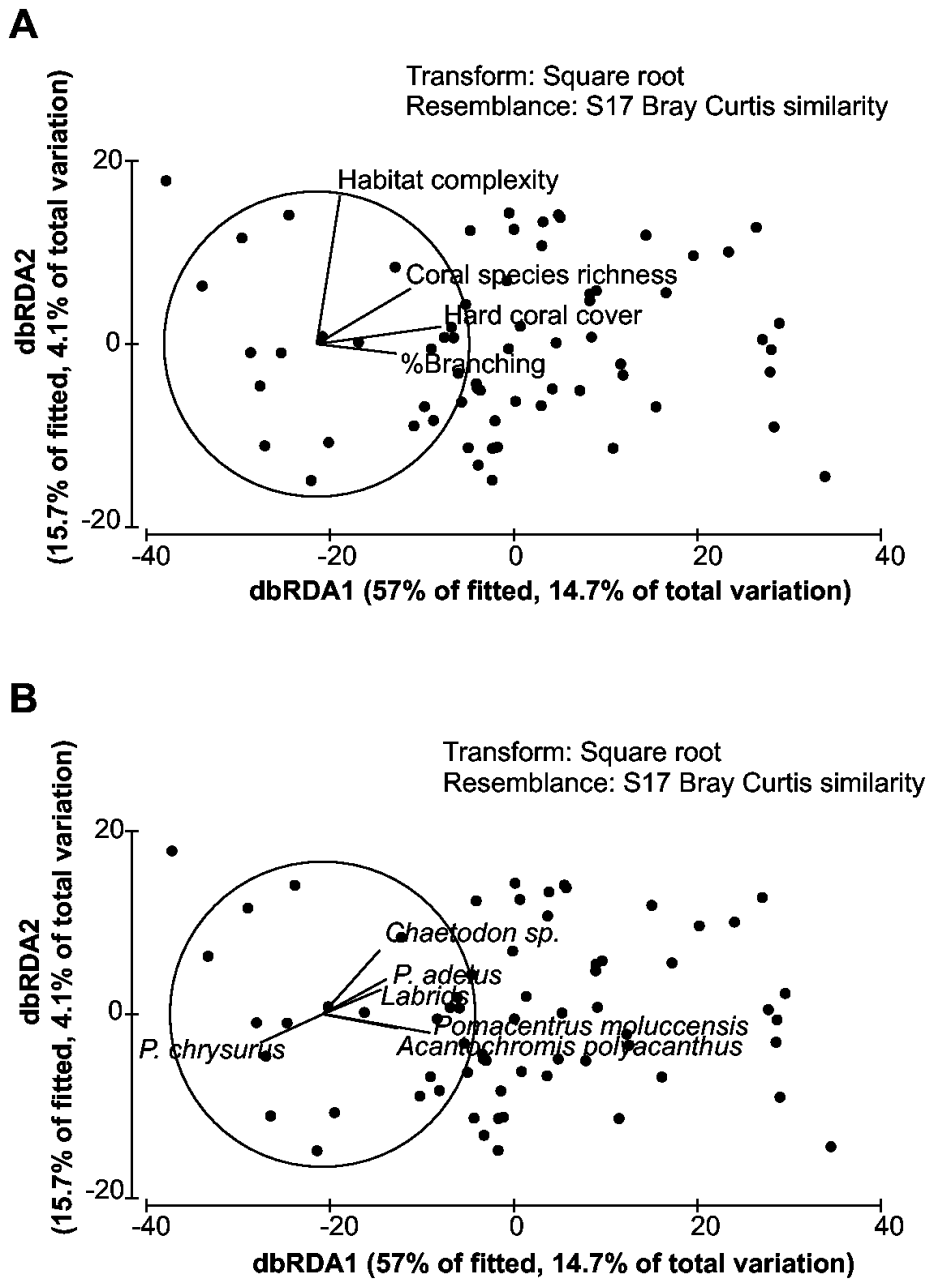
Fish community structure. Constrained distance-based redundancy analysis plot of fish community fitted to significant predictor variables (hard coral cover, habitat complexity, coral species richness, branching coral cover) identified using forward DistLM selection procedure and AIC selection criteria (Table 4). The relative influence of fitted predictor variables is indicated by the length of vector overlays. Each black point represent a single sample. **a**. Fitted predictor variables. Significant variable are identified with an asterisk (*) **b**. Fish species vectors.

Distance-based linear models revealed qualitatively similar relationships between habitat variables and the abundances of the 19 most abundant coral reef fish taxa to those described by regression trees, except that habitat complexity was also identified as a significant variable (Table 4, Figure 6). Habitat variables explained 25.8% of the variation in the fish community structure (Table 4). Percent hard coral cover was the most important predictor variable in the environmental data set, explaining just under 10% of the variability in the fish abundance (Table 4). Habitat complexity, coral species richness and percent branching coral cover were also identified as important predictor variables, explaining an additional 10.7% of variation in fish community structure (Table 4). Other measured variables did not improve the model significantly (Table 4).

**Table 4:** Proportion of variance in fish taxa abundances explained by substratum predictor variables in forward DistLM.

**Variables**	**Sequential tests**				
	**SS**	**Pseudo-F**	**p**	**Prop.**	**Cumul.**
**Hard coral cover (%)**	**11986**	**7.02**	**0.000**	**0.099**	**0.099**
**Habitat complexity**	**4801.3**	**2.9**	**0.005**	**0.04**	**0.138**
**Coral species richness**	**4501.7**	**2.79**	**0.006**	**0.037**	**0.176**
**Branching coral cover (%)**	**3664.4**	**2.32**	**0.017**	**0.03**	**0.206**
Coral diversity	2445.5	1.56	0.136	0.02	0.226
Plate coral cover (%)	2045.6	1.31	0.239	0.017	0.243
Habitat diversity	1813.4	1.17	0.335	0.015	0.258

(Prop. is the proportion of variance explained by the predictor. Cumul. is the cumulative proportion of variance explained by sequential predictors). Significant predictors indicated in bold.

Fish community structured differed with increasing hard coral cover, habitat complexity, coral species richness and percent branching coral cover (Figure 6a). Specifically, *Chaetodon* *sp., Pomacentrus adelus, P. moluccensis, Acanthochromis polyacanthus* and several species of Labridae were associated with high levels of hard coral cover, coral species richness, habitat complexity and branching coral cover (Figure 6a,b). Habitat complexity was identified as particularly important variable in determining *Chaetodon* *sp.* abundances. On the other hand, *P. chrysurus* was associated with low levels of hard coral cover, habitat complexity, coral species richness and branching coral cover (Figure 6a,b). *Chaetodon* *sp.* exhibited stronger associations with high habitat complexity in comparison to other fish species studied. *P.adelus* and labrids appeared to be more abundant in areas with high coral species richness, while higher abundances of *A.polyacanthus* and *P.moluccensis* were associated with high live coral cover, branching coral cover in particular (Figure 6a,b).

## Discussion

The role of topographic complexity and coral cover has dominated the literature on the importance of habitat structure to reef fish communities and their response to disturbance (see reviews by [19,23,60,61]). While various measures of habitat diversity have also implicated the importance of the variety of substrata [5,7,8,33], the relative effects of these potentially interacting factors have been little studied. Here we have shown that for small site-attached fish species in Lizard Island lagoon, coral species richness is the best predictor of reef fish diversity, with coral cover of some significance, and topographic complexity much less so. Coral richness was also implicated in explaining total reef fish abundance and changes in species composition, although to a lesser extent. Coral cover had important effects on reef fish diversity, abundance and species composition, especially below levels of 20%, with few species having preferences for areas of low coral cover and low coral species richness.

Coral species richness appears to play a more important role than habitat complexity in explaining species richness in this reef fish community, possibly due to a high level of coral dependence and habitat specialisation. At least 9-11% of coral reef fish are dependent on live coral [9,19], although some families appear to have a much higher percentage of species dependent on live coral than others (e.g. 53% Pomacentridae [62]). Furthermore, juveniles of many fish species prefer to settle near live coral [9]. Reasonably high levels of specialisation have been reported for at least some coral reef fish families (e.g. [7,10,44,62]). Although some species of fish tend to have similar preferences for specific coral species or morphologies that provide fitness advantages [28,46,47,63], there are also differences among species in the types of corals they inhabit or prefer to feed on [34,64,65]. Therefore, a higher diversity of coral species should provide a greater range of habitats for habitat specialist fishes. Coral species also vary greatly in their physical structure and presumably a larger number of corals would provide a greater diversity of shelter sites that could be exploited by a wide range of species.

Further studies are now needed to determine if some coral species are favoured over others by a broad range of reef fishes and if habitat specialisation among coral species could explain the coral species richness – fish species richness relationship observed here. Messmer et al. [66] recently demonstrated, by constructing small patch reefs with different coral assemblages, that reefs of higher diversity generally supported more species. However, they showed that the effects of coral diversity were explained by the inclusion of certain, highly preferred coral species. It is the loss of coral species preferred by the greatest range of fish species that will have the greatest impact on the fish assemblage. An extreme example may be the cardinalfishes, many of which show the same strong preference for a single coral species with a particular growth form [44].

Percent hard coral cover was also identified as an important predictor of fish community structure. Similar to reports from some other studies (e.g. [8,9,15,41,60]), fish species richness and abundance increased with hard coral cover. Coral cover had a greater influence on fish abundance than on fish species richness, which might be expected because higher coral cover increases habitat area for coral-associated fishes, while not necessarily increasing the range of habitat types available. Changes in fish community structure became most apparent as coral cover fell below 15-20%, with such quadrats and sites exhibiting lower fish species richness and abundance. These thresholds for the decline in coral cover and its effects on the richness of fish communities are consistent with the findings of Wilson et al. [20], who suggested that fish community richness should be expected to increase with coral cover until coral cover reaches approximately 20%. Wilson et al. [60] reported declines in the abundance of 62% of coral-dwelling fish species with declines in coral cover as little as 10%, however the greatest declines in fish species richness occurred when coral cover fell below 20%. The potential relationship between coral species richness and coral cover may make it difficult to identify which one of these habitat characteristics were responsible for the changes in fish community reported in these previous studies. Assessing the relative amounts of variation explained by these factors requires that both are quantified within the same study, as we did here.

In the lagoon of Lizard Island, percent hard coral cover had a weak but significant positive relationship with coral species richness. Therefore, it is reasonable to assume, that a reduction in hard coral cover would be associated with some reduction in coral species richness. The regression tree approach enabled us to partition the effects of different habitat variable on fish community to some degree. Fish species richness showed a stronger positive relationship with coral species richness and fish abundance had a stronger relationship with percent hard coral cover. This suggests that the abundances of some common fish species are driven by the availability of particular coral species. However, at this stage it is not possible to identify the precise mechanisms involved.

Even though habitat complexity did not appear to influence overall fish species richness and abundance in this survey when all variables where considered together, a weak but significant positive relationship was found between fish species richness and habitat complexity when just these two variables were examined. Moreover, habitat complexity was one of the 4 significant predictors of the relative abundance of the 19 most commonly observed fish taxa. Since habitat complexity, coral species richness and hard coral cover were weakly inter-correlated, it is reasonable to assume the reduction in one trait could potentially cause some reduction in another. It has been suggested that reduction in topographic complexity is not necessarily a direct consequence of massive reduction in live coral cover [19] and in many areas dead coral skeleton may remain undisturbed, providing foundation for new coral growth (e.g. [67]). In other areas, mainly dominated by erect branching corals, after death of live coral tissue, fragile skeleton can be exposed to destructive weathering and wave action [68] and reduction in habitat complexity can be rapid [18,69–71], causing loss of shelter sites. The potential inter-relationships between habitat complexity, hard coral cover and coral species richness makes it difficult to separate the specific effect of each variable on the structure of the coral reef fish community. 

Habitat appeared to not only influence fish species richness and abundances, but also species composition. Hard coral cover, habitat complexity, coral species richness and branching coral cover were identified as important variables in determining fish community structure. Only *P. chrysurus* appeared to exhibit a preference for areas with low coral cover, habitat complexity and coral species richness. The majority of fish species occupied areas of high coral species richness, habitat complexity and/or coral cover, with some having slightly stronger preference for areas with high coral cover (e.g. *A. polyacanthus* and *P. moluccensis*), and some having relatively stronger positive relationships with high habitat complexity (e.g. *Chaetodon* *sp.*). The results of our study support earlier findings by Holbrook et al. [72] and Wilson et al. [20], who reported that coral cover has the greatest effect on coral-dependant fish communities when coral cover remains below 10%. 

Small fish species, which remain close to the substratum, might be expected to be influenced the most by the substratum characteristics [33,73]. Smaller fish species would be expected to be more vulnerable to predation and the availability of a range of shelter sites might have fitness advantages for these species [28,74,75]. Damselfish species are expected to have close associations with habitat structure due to their site attached behavior and many species are suggested to use corals for refuge sites [6,26,62,76]. Indeed, positive relationships of most damselfish species with both coral species richness and hard coral cover were observed. In contrast, larger and more mobile species are less likely to exhibit close association with coral species richness or coral cover. Correlations between labrid abundance and coral species richness and/or live coral cover, reported by some other studies [5], have been suggested to be largely driven by behavioral traits, such as social interactions with other fishes [77], or dependence on the prey distribution, which may have strong associations with the availability of living corals [5].

Our results support a hypothesis that both coral species richness and hard coral cover are critical to the maintenance of the local diversity of small site-attached reef fishes. Consequently, we should expect that loss of coral species or reduction in coral cover will have a significant impact on fish communities and these factors must be distinguished in future studies. We predict that thresholds of habitat decline at which the largest effects on fish community and the greatest potential loss of fish biodiversity can be expected are when coral species richness falls below 8 species and/or hard coral cover falls below 10%. Loss of coral species may be an important factor in explaining the close relationship between declining fish diversity and declining coral cover that has been observed in some regions (e.g. [9]). Moreover, coral cover may play an important role in supporting more abundant fish communities, while high coral species richness enhances fish diversity. Longer-term effects of declining topographic complexity may be less important in this system as has been described elsewhere (e.g. [18]), but may be more important for larger, more mobile reef fishes. Further multi-factorial and long-term studies in different reef habitats are necessary to complete the picture of the nature of the relationship between fish and their complex and fragile habitat. 
